# A Study on the Relationships between Water Film Thickness, Fresh Properties, and Mechanical Properties of Cement Paste Containing Superfine Basalt Powder (SB)

**DOI:** 10.3390/ma14247592

**Published:** 2021-12-10

**Authors:** Hengrui Liu, Zhenghong Tian, Haoyue Fan

**Affiliations:** 1 College of Water Conservancy and Hydropower Engineering, Hohai University, Nanjing 210098, China; 171302020037@hhu.edu.cn (H.L.); zhtianhhuu@163.com (Z.T.); 2 State Key Laboratory of Hydrology Water Resources and Hydraulic Engineering, Hohai University, Nanjing 210098, China

**Keywords:** superfine basalt powder (SB), water film thickness (WFT), fresh properties, compressive strength, mathematical empirical model

## Abstract

In this paper, the effect of a newly developed superfine basalt powder (SB) on the fresh and mechanical properties of cement paste was studied. The concept of water film thickness (WFT) was cited to explain the influence of SB on fresh and mechanical properties and related mathematical model formulas were established. In addition, the relationship between the fresh properties and mechanical properties of paste was also explored. The results indicated that SB can improve the segregation resistance and cohesiveness. The maximum improvement rate relative to the control cement paste was 75.4% and 50.4%, respectively. The 5% SB and 10% SB reduced the fluidity in the range of 4.1–68.7% but increased the early and late compressive strength in the range of 1.2–25.7% compared to control cement paste under different water/cementitious materials (W/CM) ratios. However, the influence of 20% SB on fluidity and compressive strength was opposite to the above behavior, and the increase rate and decrease rate were 1.8–11.8% and 1.1–13.9% respectively. The WFT was the most important factor that determined the compressive strength, rheological parameters, and flow parameters of paste containing SB, while the substitute content of SB and WFT together determined the bleeding rate and cohesiveness. Among them, the correlation between bleeding rate and WFT increased with time. The empirical mathematical models between WFT, fresh properties, and compressive strength were established and verified by other mineral admixtures, which were successfully extended and applied to the entire field of cement-based materials.

## 1. Introduction

Cement has become the most common building material in engineering with the increasing global demand for construction, but the manufacturing process of cement causes serious environmental pollution and energy consumption [1,2,3]. Therefore, the development of green supplementary cementitious materials has been the focus of research in the field of cementitious materials, which can improve the performance of mixtures while saving costs and protecting the environment [4,5]. In recent years, some supplementary cementitious materials (SCM), such as silica powder, fly ash, zeolite, and limestone powder, have been used to substitute part of cement, but it is far from meeting the actual requirements of engineering applications [6,7,8,9,10]. It is a meaningful and promising topic to develop new green supplementary cementitious materials in the field of engineering construction.

Basalt has been used in engineering as a new type of cementitious materials due to its good properties. Weidong et al. [11] found that stone matrix asphalt (SMA) mixtures containing basalt coarse aggregate and fine aggregate had good rutting resistance, but poor resistance to cracking and moisture susceptibility. Dobiszewska et al. [12] reported that mixing basalt powder into concrete can improve flexural and compressive strength. P.P. Li et al. [13] found that coarse basalt aggregates slightly reduce the mechanical properties of UHPC. Ponzi [14] used basalt powder (BP) to Carbon Capture and Storage (CCS) wells, proving that low BP content and high water–binder ratio can improve the paste’s resistance to CO_2_ degradation, reduce low porosity, and provide suitable mechanical properties. Matykiewicz [15] reported that increasing the replacement content of basalt powder can improve the thermomechanical properties of epoxy composites. Laibao et al. [16] proved through microscopic tests that basalt powder can consume calcium hydroxide in cement paste and has good pozzolanic reactivity. Moreover, the basalt powder also increases the consistency and retardation time. Saraya [17] also found that basalt powder had low pozzolanic activity in the early stage and increased with time, and that the improvement effect of basalt powder on the mechanical and physical properties was better than that of limestone. Kostrzewa-Demczuk [18] found that, in autoclaved bricks, the sample containing 10% basalt powder and 10% water has high compressive strength, high sealing performance, and medium water absorption, which meets the requirements of wall construction materials. The above results indicate that there are many studies on the microscopic and mechanical properties of basalt powder, but there has been little exploration on the fresh properties of basalt powder. The study of fresh properties is particularly important in cement-based applications, such as self-compacting concrete and grout materials, where excellent rheological properties are a prerequisite for effective application of paste. In addition, the adhesiveness and segregation resistance of basalt powder to paste also need to be studied.

The water film thickness (WFT) is an effective means to characterize fresh properties. The mechanism of WFT is to quantitatively characterize the surface effect and filling effect of the material simultaneously, which indirectly represents the particles’ spacing [19]. Since the added water must first fill the voids between the solid particles, and the water in the voids cannot be used for lubrication, the fluidity of the paste can be effectively increased only by exceeding the free water required to fill the voids. The ultra-fine mineral admixtures can effectively reduce the voids of the mixture, which indicates that more free water can be used for lubrication to enhance fluidity. At the same time, the addition of ultrafine admixtures increases the specific surface area, requiring more free water to coat the particles. The WFT is a manifestation of the net effect of particle filling and surface effects. WFT has been gradually applied in the field of cement-based materials in recent years, and the relationship between WFT and the fresh properties has been established in different scenarios [20,21,22,23,24,25]. Therefore, in this paper, WFT was used to characterize the fresh properties of cement paste containing ultrafine basalt powder. In addition, the relationship between the different fresh properties were also explored.

In this paper, the fresh properties of cement paste containing a newly processed superfine basalt (SB) were explored, and the WFT is used to characterize the effect of SB on the fresh properties. The following four points were the main contributions of this paper:

(1) The effects of SB on the macroscopic fluidity, microscopic rheology, segregation resistance, cohesiveness and compressive strength of paste were studied.

(2) The influence of WFT on the fresh properties was studied, and related mathematical models were established.

(3) The relationships between different fresh properties of cement paste containing SB were also explored, and related mathematical models were established.

(4) The mathematical models established were verified in other mineral admixtures, providing theoretical guidance for the application of mineral admixtures in cement-based materials.

## 2. Materials and Methods

### 2.1. Materials

**Figure 1:**
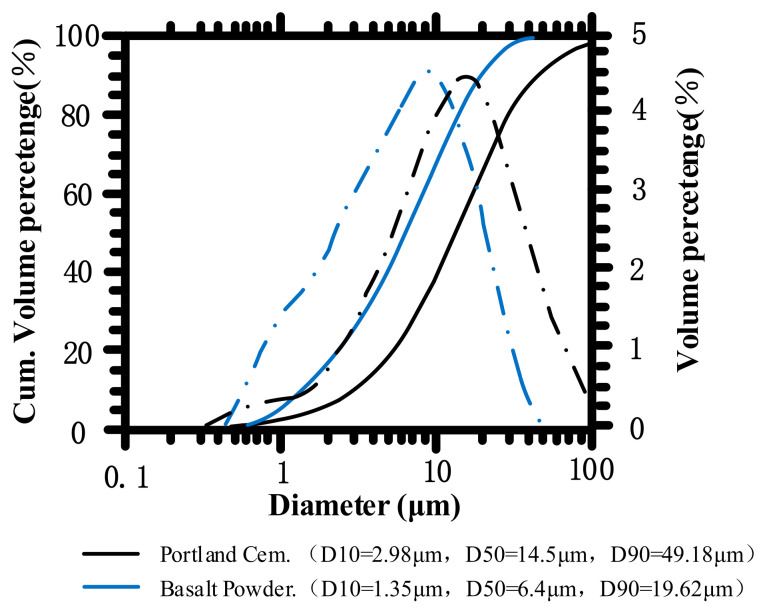
Particle size distributions of OPC and SB.

**Figure 2:**
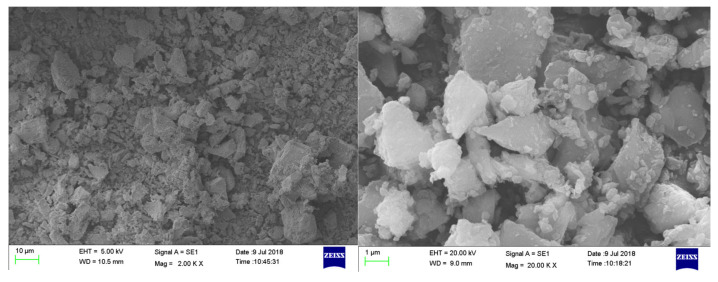
SEM morphology of SB.

Two types of cementitious materials were used in this study: superfine basalt powder (SB) (Beijing Basalt Stone Material Factory, Hebei, Baoding, China) and ordinary Portland cement (OPC) (Anhui Conch Cement Company, Anhui, Wuhu, China). The OPC with a strength grade of 42.5 was selected, which conforms to the Chinese standard GB175-2007. The SB was a mineral powder mined from the Chengde basalt vein and processed by ultrafine grinding. The chemical composition of SB and OPC is given in Table 1. The composition of SiO_2_, Al_2_O_3_, and Fe_3_O_4_ in SB was more than 70%, which has a good volcanic ash effect. The SB met the requirements of mineral admixtures for use in the field of cement-based materials according to ASTM C 618. The particle size distribution of SB and OPC is shown in Figure 1. The particle sizes of SB and OPC are 6.4 μm and 14.5 μm, respectively. The specific surface area corresponding to SB and OPC was calculated to be 649.5 m^2^/kg and 367.9 m^2^/kg, respectively. Figure 2 presents the Scanning Electron Microscope (SEM) (Carl Zeiss AG, Oberkochen, Germany) photography of SB. The SB had an irregular prismatic shape and did not have a lubricating effect. It mainly affected the mechanical and fresh properties of paste through filling effects and surface effects. 

### 2.2. Experiment Plan

In this study, SB replaced cement with 5%, 10%, and 20% mass percentages, and the properties were tested at water/cementitious materials (W/CM) ratios of 0.5, 0.55, 0.6, 0.65, and 0.7, respectively. The properties tested include void ratio, packing density, water film thickness (WFT), flow rate, flow spread, apparent viscosity, yield stress, bleeding rate, cohesiveness, and compressive strength. Since this was to investigate the effect of SB on the fresh properties and mechanical properties of paste, no superplasticizer was added here in order to avoid confusion. All experimental tests were performed in a laboratory at 20 ± 1 °C and a humidity of 70%.

### 2.3. Test Methods

#### 2.3.1. Flow Rate and Flow Spread

Marsh cone (Tianjin Changji Testing Instrument Technology Company, TianJin, China) and mini-slump cone (Tianjin Changji Testing Instrument Technology Company, TianJin, China) were used to test the fluidity of cement paste. The mini slump cone version proposed by Ouchi and Okamura [26] was used to test the flow spread. The mini-slump cone had a top diameter of 70 mm, a bottom diameter of 100 mm, and a height of 60 mm. The flow rate was measured by the marsh cone test according to the JC/T 1083-2008 standard. 

#### 2.3.2. Yield Stress and Apparent Viscosity

The NXS-11A coaxial rotational viscometer (Shanghai Precision Instrument Company, Shanghai, China) was used to measure the apparent viscosity and yield stress of cement paste [27]. Based on the interval of rheological parameters tested, it was necessary to use both the A system and the B system of the viscometer to conduct the test. The diameter and height of the rotors of the A system were 3.8 cm and 7 cm, while the diameter and height of the rotor of the B system were 3.1 cm and 5 cm, and the diameter of the outer cylinder was 4 cm. Through System A and System B, the corresponding shear stress of paste at different shear rates can be measured. Here, the improved Bingham model was used to fit the test data points, and the apparent viscosity and yield stress can be obtained. According to the Equation (1):

τ = τ_0_ + μ × γ + c × γ^2^ (1)

where γ is the shear rate, μ is the apparent viscosity, τ_0_ and τ are yield stress and shear stress, respectively.

#### 2.3.3. Cohesiveness

The cohesiveness of the cement slurry was tested by the mini-sieve separation experiment [28]. The sieve mesh (Anping Youdun Metal Wire Mesh Products Company, Hebei, Anping County, China) used in this study has an aperture of 0.6 mm. First, the mass of the sieve and the mass of the beaker (Anping Youdun Metal Wire Mesh Products Company, Hebei, China) used to receive the cement paste were measured, which were m_1_ and m_2_, respectively. About 400 g of cement paste was weighed into the beaker, and the beaker was slowly poured onto the sieve from a height of 300 mm. Part of the cement paste can fall into the bottom beaker through the sieve. After the cement paste no longer dripped from the sieve, the mass of the sieve and the mass of the beaker were reweighed to m_3_ and m_4_ respectively.

According to Equation (2), the sieve separation index (SSI) was defined as:

SSI = (m_4_−m_2_)/(m_3_−m_1_) (2)

The SSI is an indicator of cohesiveness of the paste. The cement paste with low cohesiveness can be dripped through the sieve, while it is difficult to drip through the sieve for the paste with high cohesiveness. Therefore, a lower SSI coefficient indicated that the paste had high cohesiveness. 

#### 2.3.4. Bleeding Rate

The cement paste was slowly poured into a 120 mm tall plexiglass container (Cangzhou Senzhong Test Instrument Company, Hebei, Cangzhou, China). After the paste level rose to a height of about 100 mm, the pouring was stopped, and the plexiglass bottle cap (Cangzhou Senzhong Test Instrument Company, Hebei, China) was sealed to prevent moisture changes. The initial surface height of the cement paste was recorded as d_0_. The height of the bleeding water surface after 1 h and 3 h was measured as d_1_ and d_2_, respectively. According to Equation (3), the 1 h and 3 h bleeding rate can be calculated by the following formula [27]:

σ_1h_ = (d_1_−d_0_)/100 σ_3h_ = (d_2_−d_0_)/100 (3)

#### 2.3.5. Compressive Strength

The compressive strength was tested in accordance with the Chinese standard GB50204-2002 [29]. The cement paste was firstly poured into a 70.7 mm × 70.7 mm × 70.7 mm mold (Monshanghong Company, Shanghai, China) and vibrated for 5 s, and then cured for 1 day in a normal temperature environment. After pouring for 1 day, the cement paste block was taken out and put into a curing room with a temperature of 20 ± 1 °C and a humidity of 90% for curing. The compressive strength test was carried out after 1, 7, and 28 days respectively, and the value of the compressive strength was taken as the average of the test results of 3 cement paste blocks. 

#### 2.3.6. Void Ratio, Packing Density and WFT

The wet filling method proposed by Kwan [30,31] was adopted to measure the void ratio and packing density of the mixture to calculate the WFT. The principle of the wet filling method is to find the maximum solid concentration of the cementitious material mixed with water as the packing density of the mixture. In this experiment, multiple cement pastes were prepared for each mixing ratio, which have the same solid particle mixing ratio but different W/CM ratios. The test range of W/CM ratio ranges from insufficient to sufficient to fill the voids between particles. The maximum solid concentration tested was taken as the packing density of the mixture. 

After the void ratio and packing density were calculated, the water film thickness (WFT) can be calculated by the following formula:

μ_0_ = μ_w_ − μ_s_ × (1 − μ_pd_)/μ_pd_ (4)

A = A_0_ × R_0_ + A_B_ × R_B_ (5)

WFT = μ_0_/A (6)

where μ_pd_ is the packing density of particles, μ_s_ is the volume of solid particles, and μ_w_ is the volume of mixing water. R_0_, and R_B_ are the volumetric ratios of OPC and SB to the total solid volume, respectively. A_0_ and A_B_ are the specific surface areas of OPC and SB, respectively.

## 3. Results and Discussion

### 3.1. The Effect of WFT on Fresh Properties of Cement Paste Containing SB

#### 3.1.1. Void Ratio, Packing Density, and WFT

**Figure 3:**
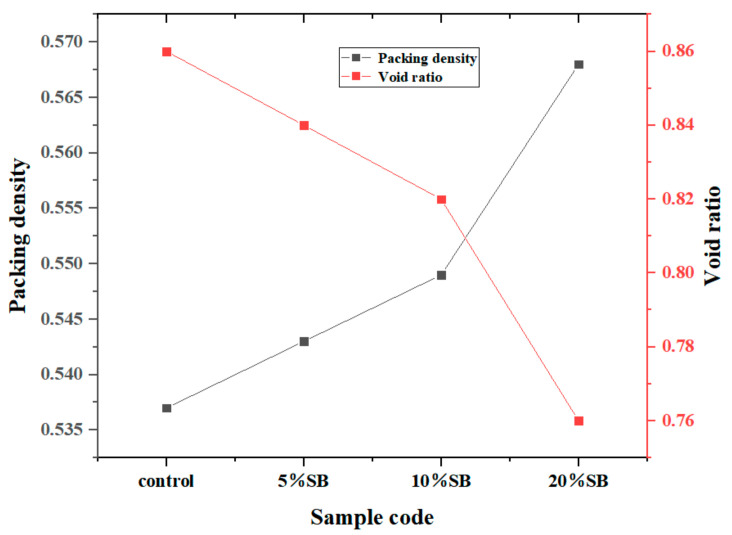
Variations of the void ratio and packing density at different SB substitutions.

The packing density and void ratio results are plotted against the SB substitutions in Figure 3. The results showed that the packing density and void ratio of cement paste increased and decreased with increasing SB substitutions, respectively. This was attributed to the filling of SB particles. Since the size of SB was much smaller than that of cement particles, it can be filled into the voids to increase a solid concentration of the mixture. Among them, the filling effect of 20% SB was the most significant, with the void ratio reduced by 11.6% and the packing density increased by 5.77%.

**Figure 4:**
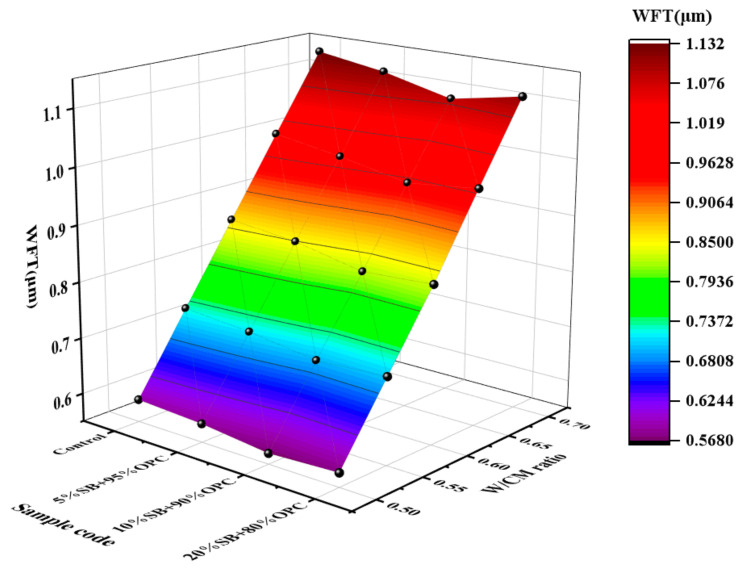
Variations of the WFT with W/CM ratio at different SB substitutions.

At the same time, the incorporation of SB also significantly increased the specific surface area of the particles, requiring more free water to wrap on the surface of the particles. The WFT results are plotted against the SB substitutions for different W/CM ratios in Figure 4. The WFT first decreased as the SB incorporation increased from 0% to 10%, and then increased as the SB incorporation further increased from 10% to 20%. This indicated that the net effects of SB particle filling and surface effects were negative at 5% and 10%, which had an adverse effect on the fluidity of the cement paste. However, it was positive only when the SB incorporation was 20%, which can play a lubricating effect and promote the fluidity of the paste. The relationship between the WFT and the fresh properties is investigated in detail below. 

#### 3.1.2. Flow Spread and Flow Rate versus W/CM Ratio and WFT

**Figure 5:**
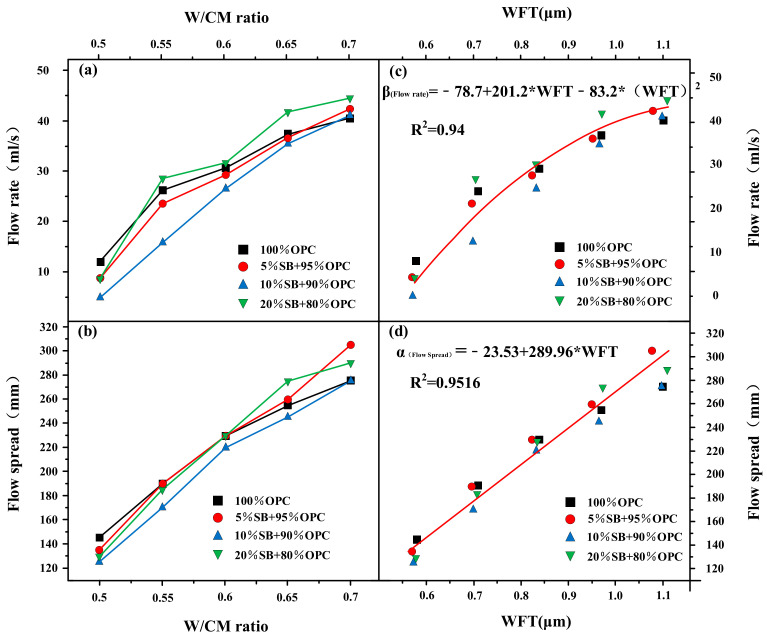
Flow rate and flow spread versus W/CM ratio (**a**,**b**) and WFT (**c**,**d**).

Figure 5a,b presents the variations of the flow rate and flow spread with the W/CM ratio for different SB substitutions. The results indicated that the effect of SB on the fluidity of cement paste was greatly affected by the W/CM ratio. At a W/CM ratio equal to 0.5, the incorporation of SB reduced the flow rate of cement paste. At a W/CM ratio equal to 0.7, the flow rate of paste containing different SB substitutions was higher than the control. In the case of other W/CM ratios, the flow rate first decreased as the SB incorporation increased from 0% to 10% and then increased as the SB incorporation further increased from 10% to 20%. Relative to flow spread, the influence of SB on the flow rate was more regular with the W/CM ratio. The flow spread exhibited the same phenomenon as the flow rate when the W/CM ratio was equal to 0.5 and 0.7. The change in W/CM ratio from 0.55 to 0.65 was more complicated. The flow spread of 10% SB was always lower than the control, while the flow spread of 5% SB and 20% SB increased from below the control to exceed the control with the increase of the W/CM ratio. 

To study the influence of WFT on flow rate and flow spread, regression analysis was performed to obtain the best fitting curve of the flow spread–WFT relationship and flow rate–WFT relationship. The flow rate and flow spread are shown as functions of the WFT in Figure 5c,d. The results indicated that the WFT, flow spread, and flow rate had excellent correlations, and R^2^ exceeded 0.9. WFT was the most critical factor to control the macro-fluidity of cement paste containing SB. The mechanism of WFT affecting the macro-fluidity of cement paste was mainly through the lubrication between particles. The increase in the WFT reduced the friction between the particles, which can improve the fluidity of the paste [32]. 

#### 3.1.3. Apparent Viscosity and Yield Stress W/CM Ratio Versus WFT

**Figure 6:**
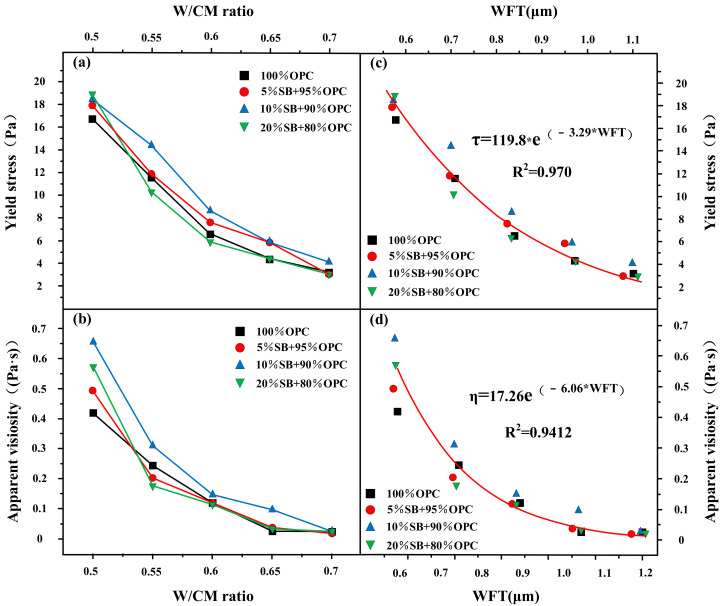
Yield stress and apparent viscosity versus W/CM ratio (**a**,**b**) and WFT (**c**,**d**).

Figure 6a,b presents the variations of the apparent viscosity and yield stress with W/CM ratio for different SB substitutions. The apparent viscosity and yield stress of 10% SB at various W/CM ratios were higher than the control, which was consistent with the variation rule of flow rate and flow spread. While the rheological parameters of 5% SB and 20% SB had complicated changes. When the W/CM ratio was greater than 0.5, the incorporation of 5% SB and 20% SB can reduce the apparent viscosity, but the decrease degree was extremely limited. For the yield stress, 5% SB and 10% SB were higher than the control at different W/CM ratios. The yield stress of 20% SB at a W/CM ratio of 0.55–0.7 were lower than the control. 

Apparent viscosity and yield stress mainly reflected the internal microstructure of the paste. This microstructure was significantly affected by the particles spacing, and WFT can indirectly represent the particles spacing. SB mainly affected the apparent viscosity and yield stress of paste by changing WFT. For example, 10% SB significantly reduced WFT, and the reduction of the particles spacing made particles more prone to collision and friction, which enhanced the shear resistance of the paste and increased the rheological parameters. 

The apparent viscosity and yield stress of paste containing SB were also significantly affected by the W/CM ratio. At a W/CM ratio equal to 0.5, different substitutions of SB could increase the apparent viscosity and yield stress to varying degrees. The different substitutions of SB had a negative effect on the apparent viscosity and yield stress.

The increase of the W/CM ratio increased the WFT of the particles within the paste, that is, increased the particles’ spacing. The rheological parameters of paste were mainly affected by colloidal attraction force, Brownian force, and gravity [33]. Among them, Brownian force and colloidal attraction force were closely related to particle spacing. The addition of SB can easily cause great changes in Brownian and colloidal gravitation at small particle spacing. On the contrary, the addition of SB had little effect on Brownian attraction and colloid attraction at large particle spacing. There was a threshold value of particle spacing affecting the rheological parameters [34]. When particle spacing exceeded this threshold, the ability of SB to influence rheological parameters of cement paste weakened. Therefore, the change of W/CM ratio can significantly affect the apparent viscosity and yield stress. 

The apparent viscosity and yield stress are shown as functions of the WFT in Figure 6c,d. It can be seen from the results that when the WFT was at a large value, SB had little influence on the rheological parameters of the paste. On the contrary, when the WFT was at a smaller value, SB plays a more significant role. Apparent viscosity and yield stress had excellent correlation with WFT, and both R^2^ values exceeded 0.94. This verifies the above conclusions; SB mainly affected the apparent viscosity and yield stress by influencing WFT. The WFT played a key role in affecting the rheological parameters of cement paste containing SB. 

#### 3.1.4. Cohesiveness Versus W/CM Ratio and WFT

**Figure 7:**
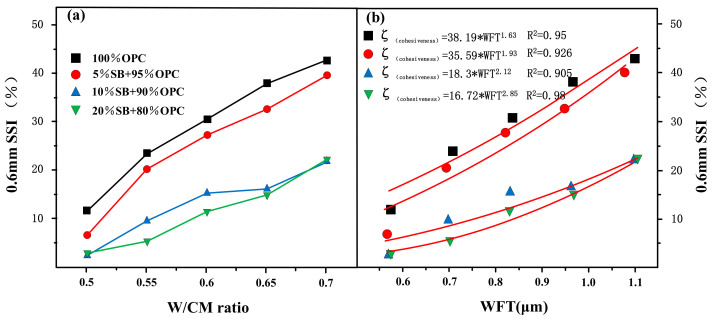
Cohesiveness versus W/CM ratio (**a**) and WFT (**b**).

Figure 7a presents the variations of the cohesiveness with W/CM ratio for different SB substitutions. The results showed that the SSI index decreased with the increase of SB substitution. The addition of SB increased the cohesiveness of cement paste. Especially when SB increased from 5% to 10%, the cohesiveness increased significantly, but when SB was further increased from 10% to 20%, the cohesiveness increased slightly. 

Cohesiveness is shown as a function of the WFT in Figure 7b. According to the results, the cohesiveness of the paste increased with increasing SB substitutions at the same WFT. However, unlike the results of flow parameters and rheological parameters, the correlation between WFT and cohesiveness was extremely poor, and the dispersion degree of data points was very large, indicating that WFT was not the only factor determining cohesiveness. As can be seen from the fitting curve in Figure 7b, it was also related to the replacement contents of SB itself. 

Cohesiveness represents the bonding strength between particles, and is related to both physical and chemical factors. When the fine SB particles filled in the voids of the cement, the contact between the particles move closer together, thereby enhancing the cohesiveness. In addition, the high specific surface area of SB also increased the electrostatic attraction between particles, thereby increasing particle aggregation. The SB contains a high content of SiO_2_ and Al_2_O_3,_ which consumed the Ca(OH)_2_ in the cement paste. Since crystalline Ca(OH)_2_ has a layered structure, the interlayer forces mainly depended on the hydrogen bonding between the oxygen atoms, and the binding forces were weak. However, the reduction of Ca(OH)_2_ content increases the bonding strength between the internal particles, which enhanced the cohesiveness of the paste [35]. The concept of WFT only considered the physical effects of SB, and did not consider the chemical effects caused by SB. Therefore, the WFT is not the only factor that determines the cohesiveness of the cement paste, but is also related to the substitute content of SB.

#### 3.1.5. Bleeding Rate versus W/CM Ratio and WFT

**Figure 8:**
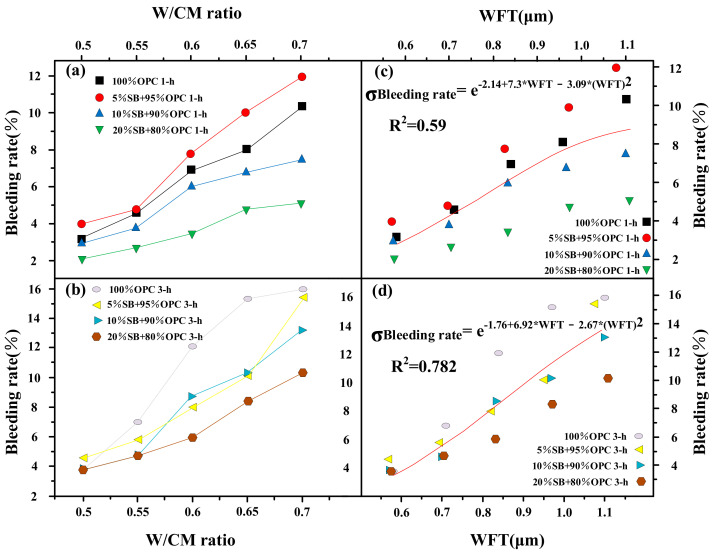
Bleeding rate versus W/CM ratio (**a**,**b**) and WFT (**c**,**d**).

Figure 8a,b presents the variations of the 1 h and 3 h bleeding rate with W/CM ratio for different SB substitutions. At 1 h, 10% SB and 20% SB reduced the bleeding rate of cement paste at various W/CM ratios, while 5% SB slightly increased the bleeding rate. At 3 h, the reduction degree of the bleeding rate by SB further increased, and the bleeding rate of cement paste containing SB was lower than the control in almost all scenarios. At a W/CM ratio equal to 0.5, 10% SB and 20% SB were reduced by 0.06% and 0.1%, respectively, based on the control. At a W/CM ratio equal to 0.7, 10% SB and 20% SB were reduced by 2.8% and 5.61%, respectively, based on the control. The above results indicated that the replacement of SB effectively reduced the bleeding rate of paste, especially in the case of high W/CM ratio and high substitution.

SB reduces the bleeding rate due to the following reasons: (i) The density of SB particles is significantly lower than that of cement particles, and they are not easy to sink under the action of water resistance. (ii) SB increases the packing density of the paste, and the close packing of the internal structure effectively prevents the sinking of the particles. (iii) SB has a high content of SiO_2_ and Al_2_O_3_, which chemically reacted with Ca(OH)_2_ to further increase the flocculation structure and internal colloid network within the paste [36].

The 1 h and 3 h bleeding rate are shown as functions of the WFT in Figure 8c,d. Similar to cohesiveness, the bleeding rate had a poor correlation with WFT, and the bleeding rate was also affected by other parameters, such as the substitute content of SB. WFT is not the only factor that determines the bleeding rate of cement paste. In addition, it was found from the Figure 8c,d that the regression coefficient R^2^ of WFT and the bleeding rate increased significantly from 1 h to 3 h, because the WFT decreased with the increase of time, and the water wrapped on the particle surface broke away from the particle and entered into the bleeding area, while the particle gradually sank into the sediment zone.

#### 3.1.6. Compressive Strength versus W/CM Ratio and WFT

**Figure 9:**
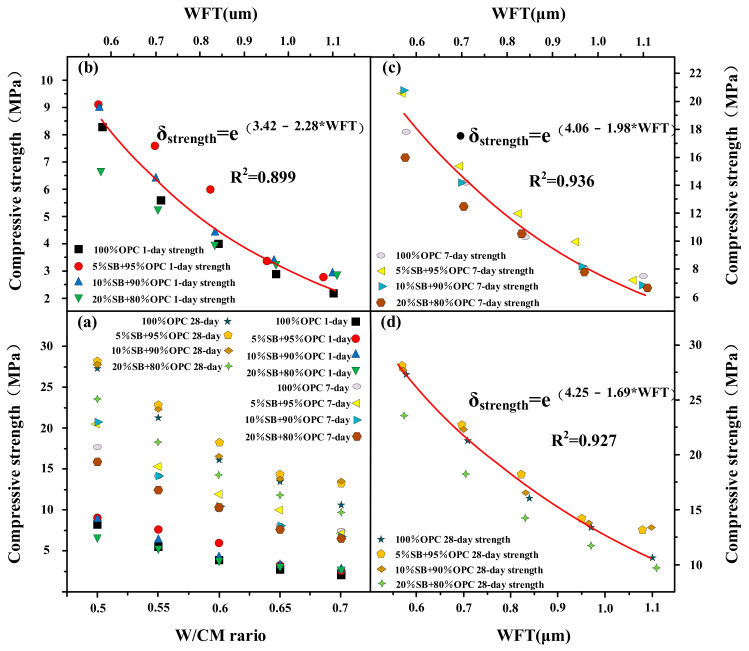
Compressive strength versus W/CM ratio (**a**) and WFT (**b**–**d**).

Figure 9a presents the variations of the 1 day, 7 days, and 28 days compressive strength with the W/CM ratio for different SB substitutions. From the results of compressive strength in different periods, both the W/CM and the replacement content of SB can affect the compressive strength of paste. At 1 day, except for 5% SB which can improve the compressive strength at low W/CM ratio, the influence of SB on the compressive strength of paste in other scenarios was not remarkable. At 7 days and 28 days, the difference in compressive strength of different cement paste samples began to appear. In general, 10% SB and 5% SB improved the compressive strength, while 20% SB reduced the compressive strength, which showed the same law under all W/CM ratios. With the increase of the W/CM ratio, the ability of SB to influence the compressive strength was gradually weakened, and the performance of SB was more effective at low W/CM ratio. 

The influence of SB on the compressive strength of cement slurry was mainly attributed to three effects: inherent characteristics, grain refinement effect, and the hydration effect [37,38]. The inherent characteristics mainly refer to the surface structure and charge characteristics of the particles. When SB was in contact with the colloidal suspension, the inherent characteristics of SB affected the nucleation and growth of hydration products, thereby affecting the compressive strength of the cement paste. The grain refinement effect can affect the interface area between SB and hydration products, making the internal structure of the paste more compact and uniform. The hydration effect was that SiO_2_ and Al_2_O_3_ inside SB consume Ca(OH)2 in paste and generate calcium silicate hydrate (CSH). The structure between SB and the cement base was more compact, which effectively reduced the void ratio and improved the mechanical properties of the colloid interfacial area [39]. But at the same time, the replacement of SB also reduced the content of the cement, which reduced the generation of hydration products. As a result, the net effect of 5% SB and 10% SB substitution was positive, while the net effect of 20% SB was negative. 

The compressive strength of 1 day, 7 days, and 28 days are shown as functions of the WFT in Figure 8b–d. The results show that the compressive strength of cement paste decreased with increasing WFT. The regression coefficient R^2^ was 0.899, 0.936, and 0.927 at 1 day, 7 days, and 28 days, respectively, indicating WFT and compressive strength have a good correlation. WFT is also the main factor that determines compressive strength because WFT can indirectly represent the distance between particles. The increase of particle spacing makes the internal structure of paste loose and increases the porosity between structures, thus reducing the mechanical properties of the colloid interface area. 

### 3.2. The Relationships between Different Fresh Properties

Section 3.1 explored the effects of SB on flow parameters, rheological parameters, cohesiveness, and bleeding rate of cement paste, and cited the concept of WFT to establish a relationship with these parameters. The mathematical models between WFT and these properties were established. This section establishes a connection between fresh properties and mechanical properties. There are two meanings of this. The relationship between the compressive strength and fresh properties of cement paste has been rarely explored so far. In addition, the empirical mathematical model with good correlation can provide references for other scholars. 

**Figure 10:**
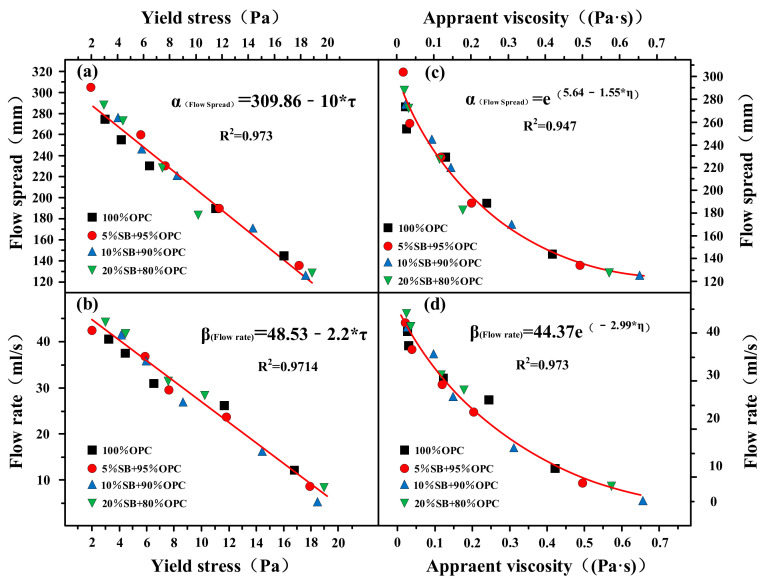
Flow rate and flow spread versus apparent viscosity (**c**,**d**) and yield stress (**a**,**b**).

The flow rate and flow spread and apparent viscosity and yield stress for SB substitutions from 5% to 20% in are shown in Figure 10a–d. The flow rate and flow spread decreased with increasing apparent viscosity and yield stress. There was a good correlation between rheological parameters and flow parameters, and the correlation coefficient R^2^ exceeded 0.95. The relationships between flow parameters and rheological parameters conformed to the following mathematical model: α_(Flow spread)_ = A + B*τ_(Yield stress)_, β_(Flow rate)_ = A + B*τ_(Yield stress)_, α_(Flow spread)_ = e^(A+B*η(Apparent viscosity))^, β_(Flow rate)_ = Ae^(B*η(Apparent viscosity))^. Flow rate and flow spread characterize the macroscopic fluidity, which is a direct manifestation of the dynamic and static flow of cement paste. The apparent viscosity and yield stress characterize the micro-rheological properties. The yield stress was the critical stress when the paste starts to flow, and the apparent viscosity was the ability of the internal structure to hinder the flow of the paste. Therefore, the yield stress and apparent viscosity can determine the flow rate and flow spread of the cement paste containing SB. 

**Figure 11:**
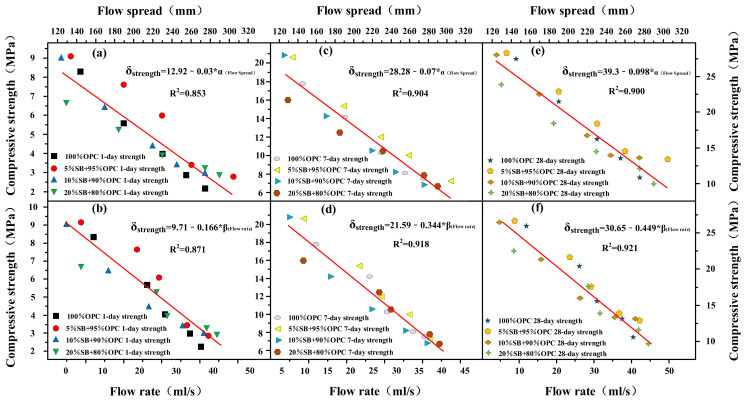
Flow rate and flow spread versus 1 day (**a**,**b**), 7 days (**c**,**d**), and 28 days (**e**,**f**) of compressive strength.

**Figure 12:**
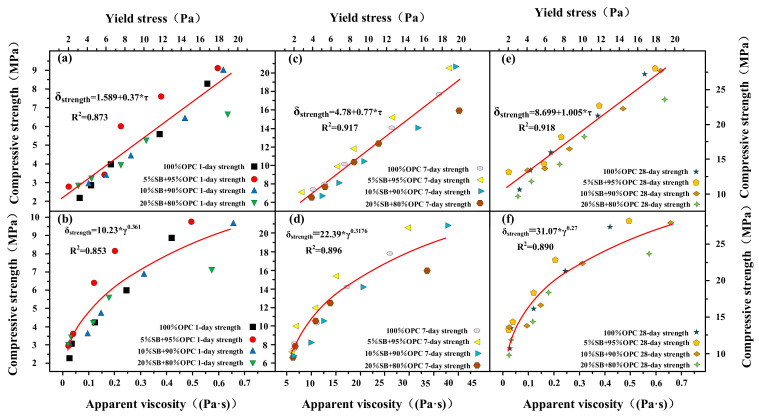
Apparent viscosity and yield stress versus 1 day (**a**,**b**), 7 days (**c**,**d**), and 28 days (**e**,**f**) of compressive strength.

The flow rate and flow spread are shown as functions of 1 day, 7 days, and 28 days of compressive strength for SB substitutions from 5% to 20% in Figure 11a–f. At 1 day, 7 days, and 28 days, compressive strength decreased with an increasing flow rate and flow spread. There was also a good correlation between compressive strength, flow rate, and flow spread. The relationship between flow parameters and compressive strength conformed to the following mathematical model: δ_(Compressive strength)_ = A + B*α_(Flow spread)_ and δ_(Compressive strength)_ = A + B*β_(Flow rate)_. The flow parameters affected compressive strength mainly through the effect of WFT and exhibits the same behavior for apparent viscosity yield stress, as shown in Figure 12. The relationship between flow parameters and compressive strength conform to the following mathematical model: δ_(Compressive strength)_ = A + B*τ_(Yield stress)_ and δ_(Compressive strength)_ = A*η_(Apparent viscosity)_^B^. At 1 day, 7 days, and 28 days, compressive strength increased with increasing apparent viscosity and yield stress. From the conclusions of the Section 3.1, it can be known that WFT was the most important factor in determining flow parameters and rheological parameters. Therefore, SB affected flow parameters and rheological parameters by influencing WFT. The change of WFT also affected the change of compressive strength, which was why the compressive strength had a good correlation with the rheological parameters and flow parameters.

**Figure 13:**
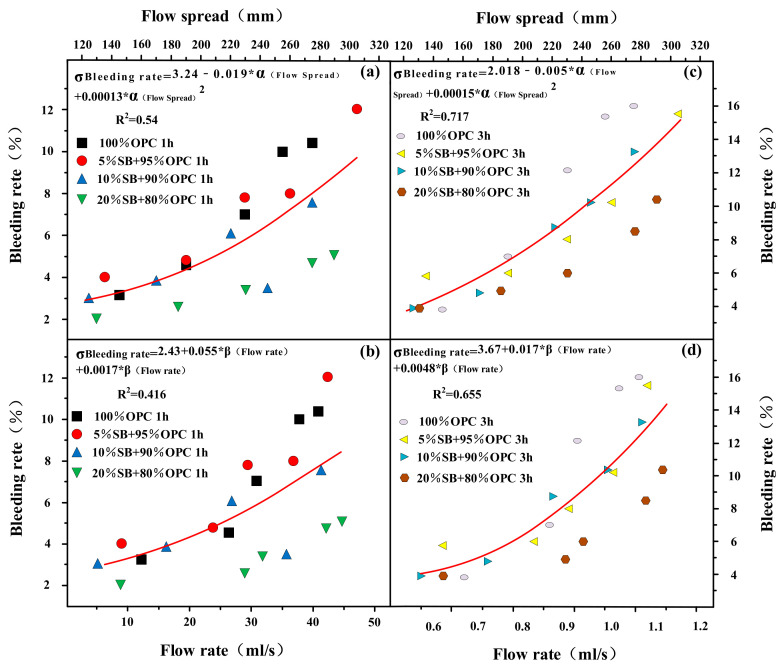
Flow rate and flow spread versus 1 h (**a,b**) and 3 h (**c,d**) bleeding rate.

**Figure 14:**
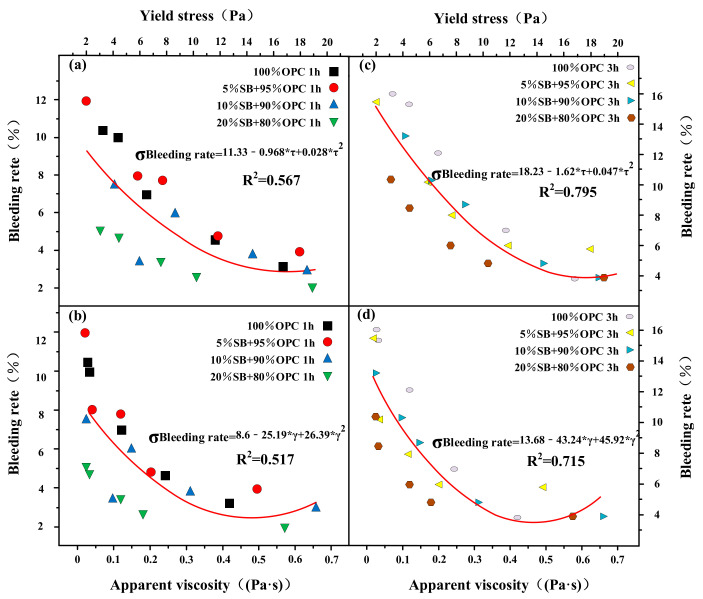
Apparent viscosity and yield stress versus 1 h (**a,b**) and 3 h (**c,d**) bleeding rate.

The flow rate and flow spread are shown as functions of the bleeding rate for SB substitutions from 5% to 20% in Figure 13a–d. The results showed that flow rate and flow spread increased with the increase of bleeding rate. However, the correlations between flow parameters and bleeding rate were poor. There were rheological parameters with the same behavior, as shown in Figure 14. Among them, the regression coefficient R^2^ was between the bleeding rate and rheological parameters, and the flow parameters significantly improved from 1 h to 3 h. This phenomenon was similar to the relationship between the bleeding rate and WFT. 

**Figure 15:**
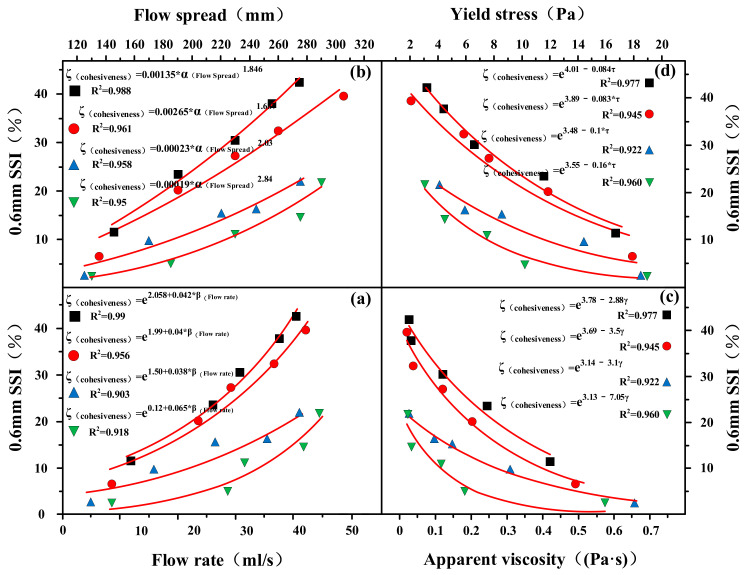
Flow rate (**a**), flow spread (**b**), apparent viscosity (**c**), and yield stress (**d**) versus cohesiveness.

The flow rate, flow spread, apparent viscosity, and yield stress are functions of the cohesiveness for SB substitutions from 5% to 20% in Figure 15a–d. The flow parameters decreased with the increase of cohesiveness, and the rheological parameters increased with the increase of cohesiveness. Neither flow parameters nor rheological parameters were the only factors that determine cohesiveness, but are also related to the substitute content of SB. 

**Figure 16:**
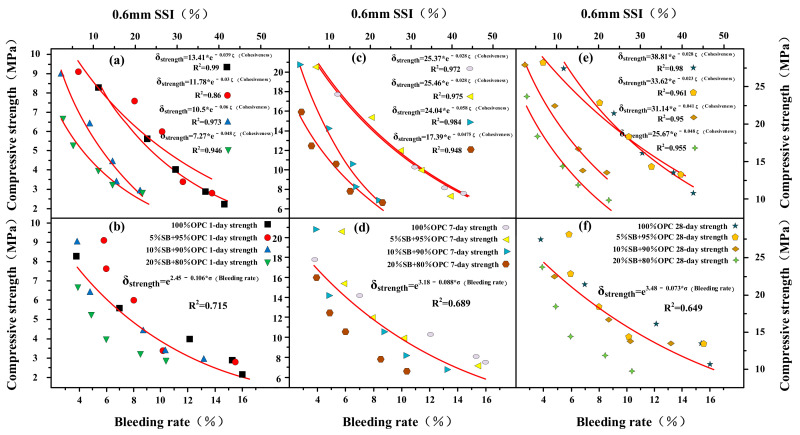
Bleeding rate and cohesiveness versus 1 day (**a**,**b**), 7 days (**c**,**d**), and 28 days (**e**,**f**) of compressive strength.

The bleeding rate and cohesiveness are shown as functions of 1 day, 7 days, and 28 days of compressive strength for SB substitutions from 5% to 20% in Figure 16a–f. The correlation between cohesiveness, compressive strength, and the bleeding rate was extremely poor. The changes in cohesiveness and bleeding rate did not significantly affect the compressive strength of cement paste containing SB.

### 3.3. Mathematical Model Validation

**Figure 17:**
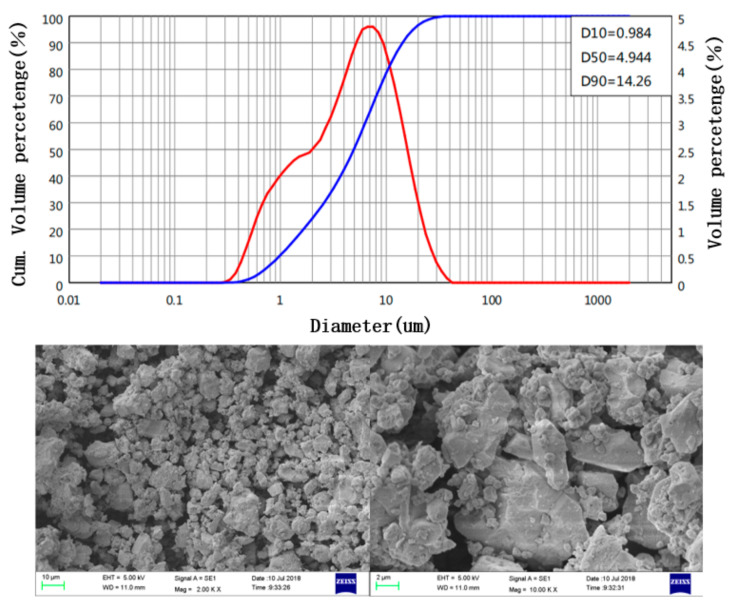
SEM photography and particle size distribution of silica powder.

The empirical models with a good relationship established in Section 3.1 and Section 3.2 are verified in this Section. The mathematical models that had a good correlation between WFT and the fresh properties were as follows: α_(Flow spread)_ = A + B*WFT, β_(Flow rate)_ = A + B*WFT + C*WFT^2^, τ_(Yield stress)_ = e^(A+B*^^WFT^^)^, η_(Apparent viscosity)_ = Ae^(B*^^WFT^^)^ and δ_(Compressive strength)_ = e^(A+B*^^WFT^^)^. The mathematical models with good correlation between the fresh properties were as follows: α_(Flow spread)_ = A + B*τ_(Yield stress)_, β_(Flow rate)_ = A + B*τ_(Yield stress)_, α_(Flow spread)_ = e^(A+B*η(Apparent viscosity))^, β_(Flow rate)_ = Ae^(B*η(Apparent viscosity))^, δ_(Compressive strength)_ = A + B*α_(Flow spread)_, δ_(Compressive strength)_ = A + B*β_(Flow rate)_. δ_(Compressive strength)_ = A + B*τ_(Yield stress)_ and δ_(Compressive strength)_ = A*η_(Apparent viscosity)_^B^. A mineral admixture (silicon powder) with a particle size and morphology similar to SB was selected here, as shown in Figure 17. The silicon powder was subjected to the same test procedure as SB with the same ratio and experimental conditions. The WFT, flow parameters, rheological parameters, apparent viscosity, yield stress, bleeding rate, and cohesiveness were obtained, and a regression analysis between these properties was performed to obtain the best fitting equation. Since it was an empirical model verification, the influence of silica fume on these properties is not described in detail here.

**Figure 18:**
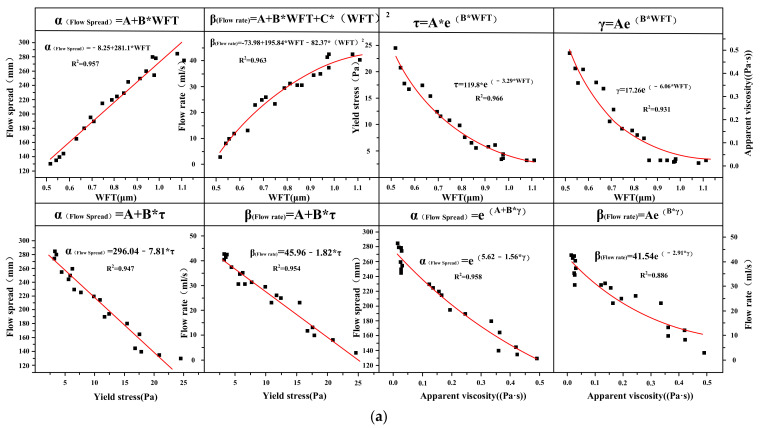

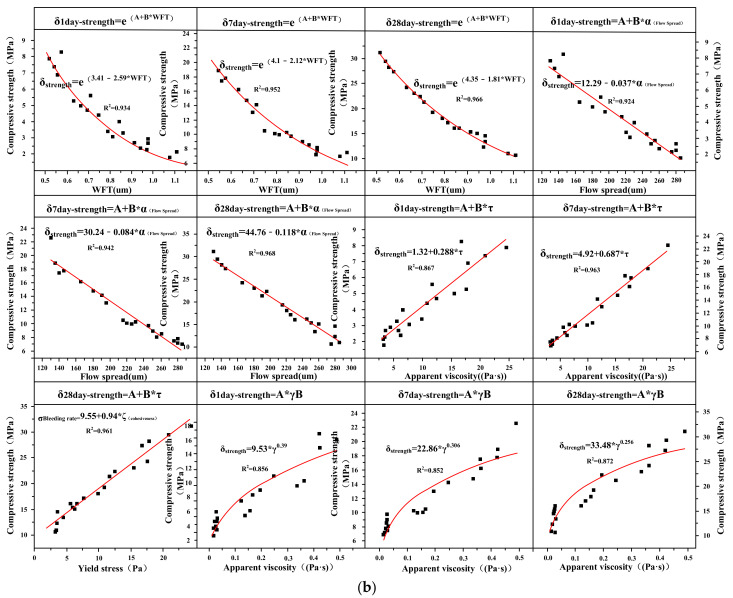
Mathematical empirical model verification (**a**,**b**): (**a**) The mathematical empirical models between WFT and fresh properties; (**b**) The mathematical empirical models between WFT, fresh properties and compressive strength.

Figure 18a,b presents the mathematical empirical model verification with the W/CM ratio for different SF substitutions. The best fitting equations of the various parameters of the cement paste containing SF were in line with the empirical models obtained in Section 3.1 and Section 3.2. All regression fitting coefficients R^2^ were greater than 0.8, and most of them were above 0.9, which proves the rationality of the mathematical empirical models obtained. The purpose of model verification was to extend and apply the empirical model of a single system of cement paste containing SB to the entire cement paste system, which can provide a reference and theoretical basis for scholars to study the relationship between different fresh properties or mechanical properties of cement paste. 

## 4. Conclusions

Based on the above results, the main conclusions of this paper are as follows:

(1) The substitution of SB can effectively improve the segregation resistance and cohesiveness of paste; the maximum improvement rate relative to the control cement paste was 75.4% and 50.4%, respectively. The 5% SB and 10% SB increased the 1 day, 7 days, and 28 days of compressive strength in the range of 1.2–25.7% but reduced the fluidity in the range of 4.1–68.7%. The 20% SB reduced the 1 day, 7 days, and 28 days of compressive strength in the range of 1.1–13.9% but increased the fluidity in the range 1.8–11.8%. The SB in the appropriate substitute content can meet the requirements of the fresh properties and compressive strength of the grouting material. As a new type of mineral admixture, SB can be used in the grouting field. 

(2) The WFT had excellent correlation with rheological parameters, flow parameters, and compressive strength, and the regression coefficient R^2^ exceeded 0.9. WFT was the most important factor that determined the rheological parameters, flow parameters, and compressive strength of cement paste containing SB. WFT and SB substitute content together determined the bleeding rate and cohesiveness. Among them, the correlation between bleeding rate and WFT increased with time. The mathematical models that had a good correlation between WFT and the fresh properties were as follows: α_(Flow spread)_ = A + B*WFT, β_(Flow rate)_ = A + B*WFT + C*WFT^2^, τ_(Yield stress)_ = e^(A+B*^^WFT^^)^, η_(Apparent viscosity)_ = Ae^(B*^^WFT^^)^ and δ_(Compressive strength)_ = e^(A+B*^^WFT^^)^.

(3) The mathematical models with good correlation between the fresh properties were as follows: α_(Flow spread)_ = A + B*τ_(Yield stress)_, β_(Flow rate)_ = A + B*τ_(Yield stress)_, α_(Flow spread)_ = e^(A+B*η(Apparent viscosity))^, β_(Flow rate)_ = Ae^(B*η(Apparent viscosity))^, δ_(Compressive strength)_ = A + B*α_(Flow spread)_, δ_(Compressive strength)_ = A + B*β_(Flow rate)_. δ_(Compressive strength)_ = A + B*τ_(Yield stress)_, δ_(Compressive strength)_ = A*η_(Apparent viscosity)_^B^. These empirical mathematical models were verified using silica fume with a similar morphology and particle size as SB, which successfully extended the empirical mathematical model to the entire field of cement-basted materials for application. 

## Figures and Tables

**Table 1:** Chemical compositions of OPC and SB used in the experiment.

Phase	Mass Percentage (%)	
	OPC	SB
SiO_2_	13.9	46.59
Al_2_O_3_	3.05	14.17
Fe_2_O_3_	4.80	14.91
CaO	72.37	10.25
MgO	1.07	4.875
Na_2_O	0.16	4.26
SO_3_	2.63	0.09
K_2_O	0.97	1.55
P_2_O_5_	0.23	0.64
TiO_2_	0.35	2.34
MnO	0.30	0.18
ZrO_2_	0.02	0.06
SrO	0.10	0.04
Cl	0.02	0.01

## Data Availability

Data Sharing is not applicable.
